# Supplementary figures and images for: Pasture intake protects against commercial diet-induced lipopolysaccharide production facilitated by gut microbiota through activating intestinal alkaline phosphatase enzyme in meat geese
Source: Front Immunol. 2022 Dec 8;13:1041070. doi: 10.3389/fimmu.2022.1041070 (PMC9774522; doi:10.3389/fimmu.2022.1041070)

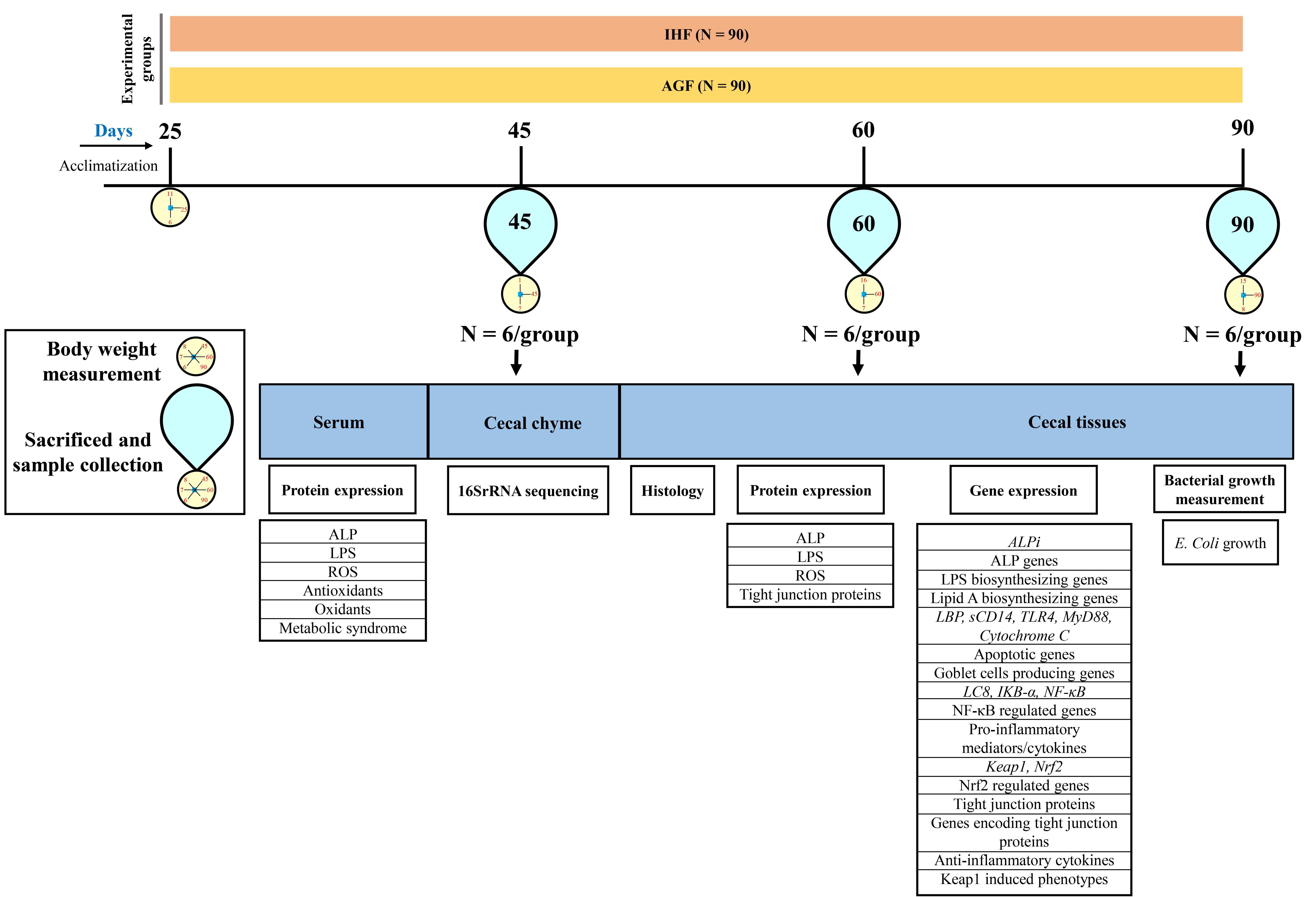

Supplement: Supplemental Figure 1 — Overview of feeding and sampling strategies. [file Image_1.jpeg]

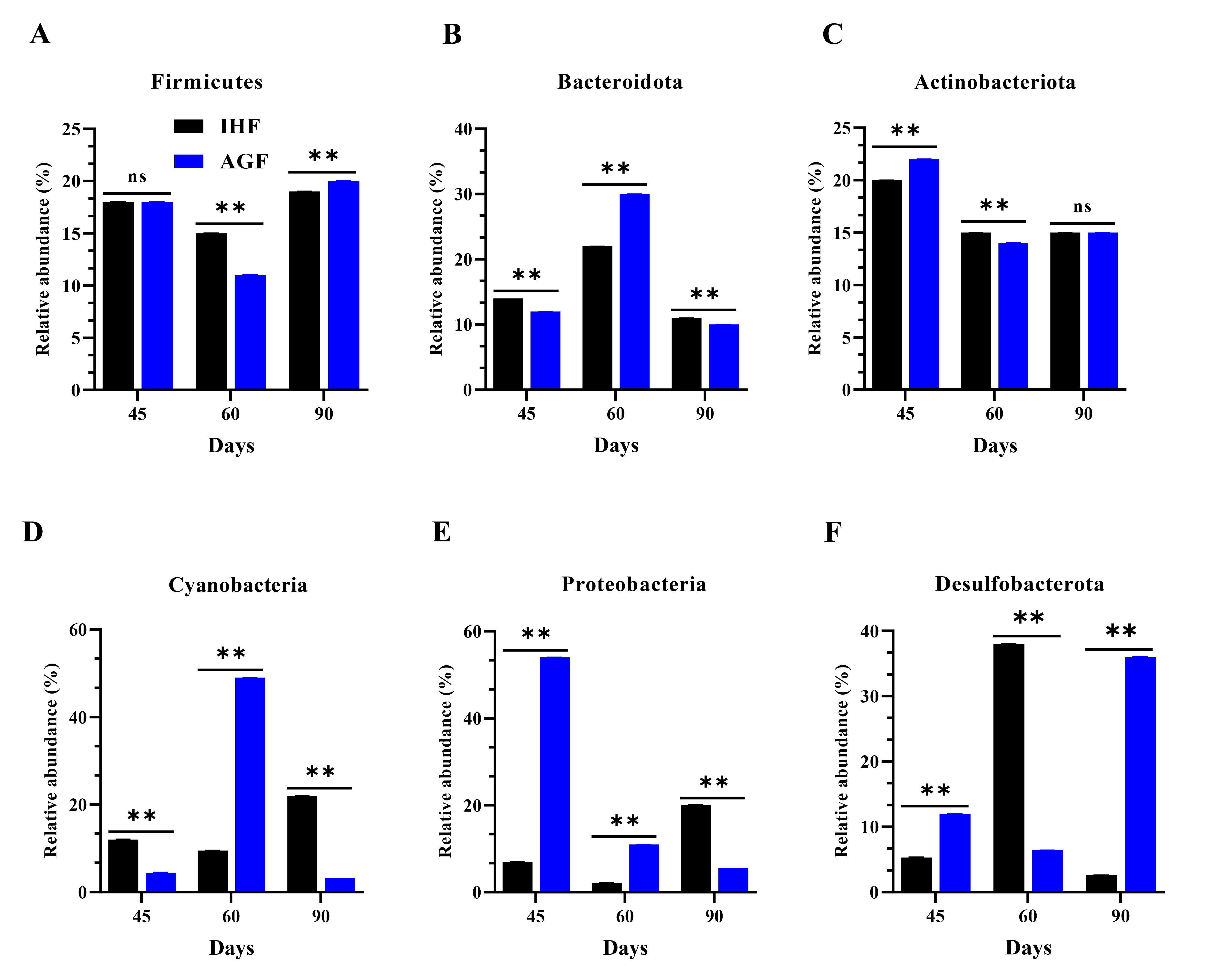

Supplement: Supplemental Figure 2 — Contributions of bacteria at phylum level to LPS biosynthesis functions. (A–F) Relative abundances (%) of the six most dominant phyla in the cecal chyme of the IHF and AGF meat geese. Data with different superscript letters are significantly different (P < 0.05) according to the unpaired student T-Test. *P < 0.05, **P < 0.01. [file Image_2.jpeg]

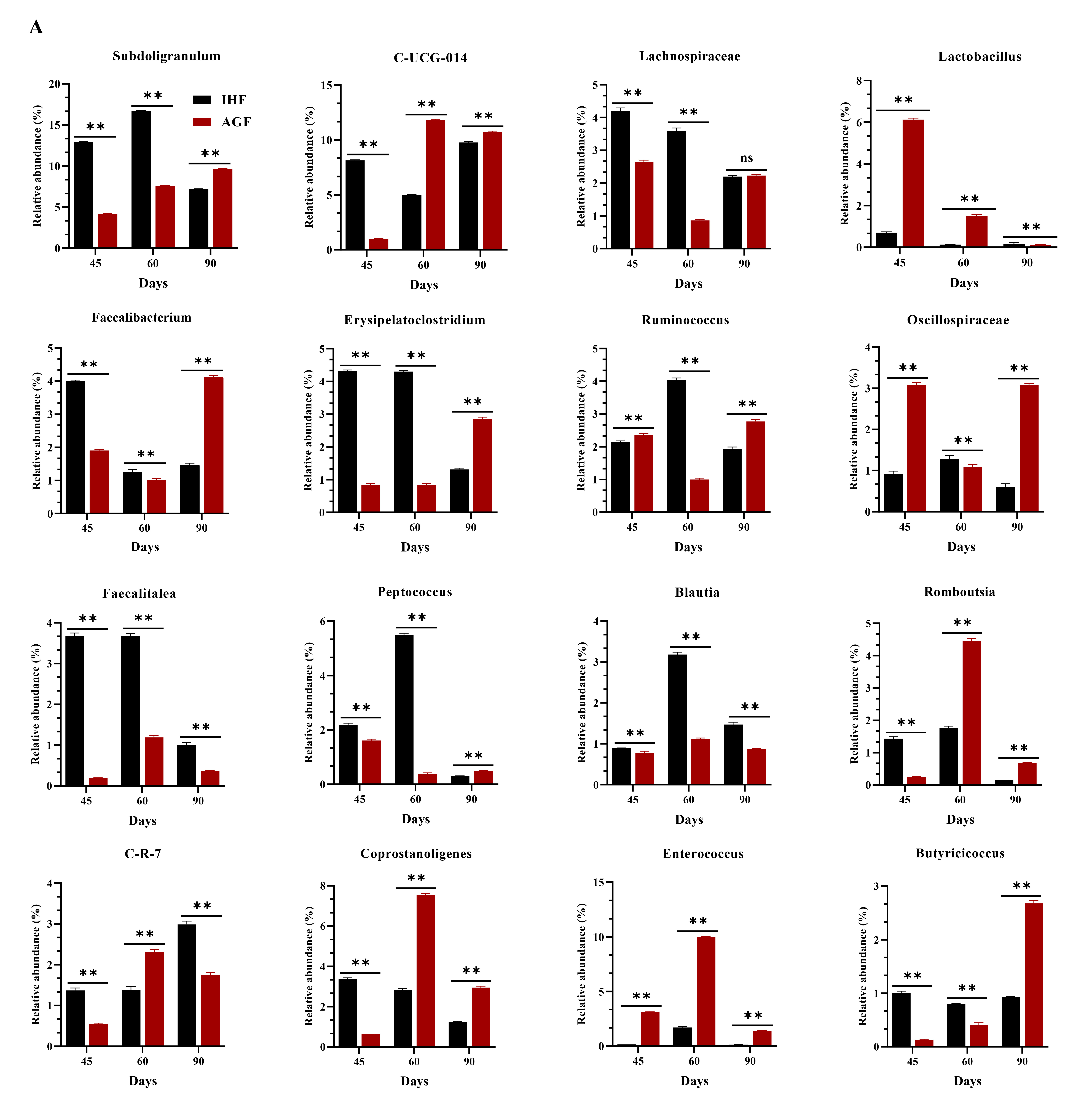

Supplement: Supplemental Figure 3 — Contribution of individual genera within each phylum to LPS biosynthesis functions. The complete name for each genus is given below. (A) Relative abundances (%) of the sixteen most dominant genera (Subdoligranulum, norank_f:norank_o:Clostridia_UCG-014, unclassified_f:Lachnospiraceae, Lactobacillus, Faecalibacterium, Erysipelatoclostridium, Ruminococcus_torques_group, unclassified_f:Oscillospiraceae, Faecalitalea, Peptococcus, Blautia, Romboutsia, Christensenellaceae_R-7_group, norank_f:Eubacterium_coprostanoligenes_group, Enterococcus, Butyricicoccus, and norank_f:norank_o:RF39) within phylum Firmicutes in the cecal contents of the IHF and AGF meat geese. (B) Relative abundances (%) of the five most dominant genera (Bacteroides, Alistipes, Parabacteroides, Prevotellaceae_UCG-001, and Rikenellaceae_RC9_gut_group) within phylum Bacteroidota in the cecal contents of the IHF and AGF meat geese. (C–E) Relative abundances (%) of the most dominant genera within phylum Actinobacteriota (Bifidobacterium), Cyanobacteria (norank_f:norank_o:Gastranaerophilales), and Desulfobacterota (Desulfovibrio) in the cecal chyme of the IHF and AGF meat geese. Data with different superscript letters are significantly different (P < 0.05) according to the unpaired student T-Test. *P < 0.05, **P < 0.01. [file Image_3.jpeg]

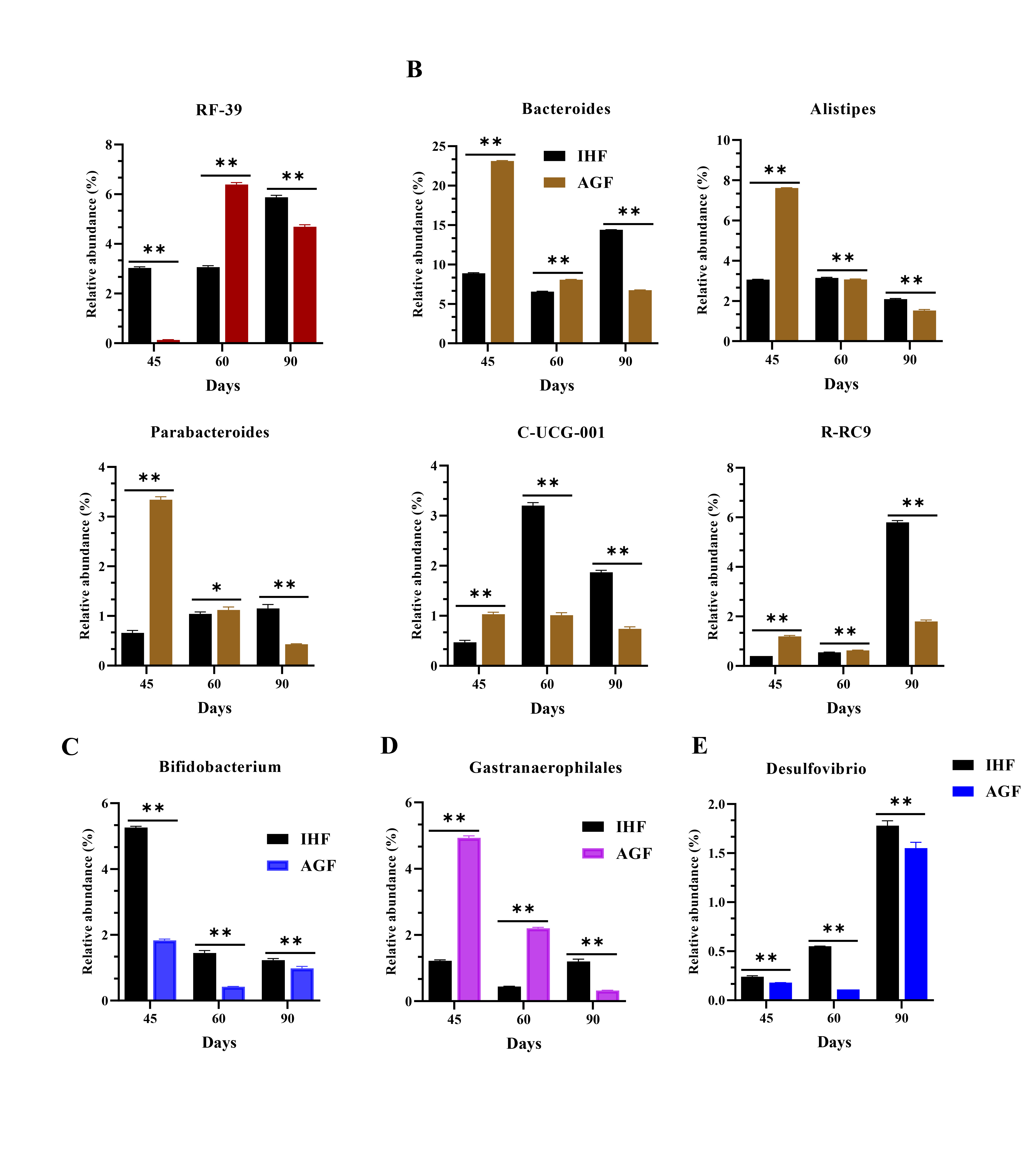

Supplement: Supplemental Figure 4 — Effect of different feeding systems on pH values of meat geese gastrointestinal tract. (A) pH of proventriculus, (B) pH of gizzard, (C) pH of ileum, and (D) pH of cecum. In-house feeding system (IHF) and artificial pasture grazing system (AGF). Data with different superscript letters are significantly different (P < 0.05) according to the unpaired student T-Test. The asterisks symbol indicates significant differences *P < 0.05, **P < 0.01. [file Image_4.jpeg]

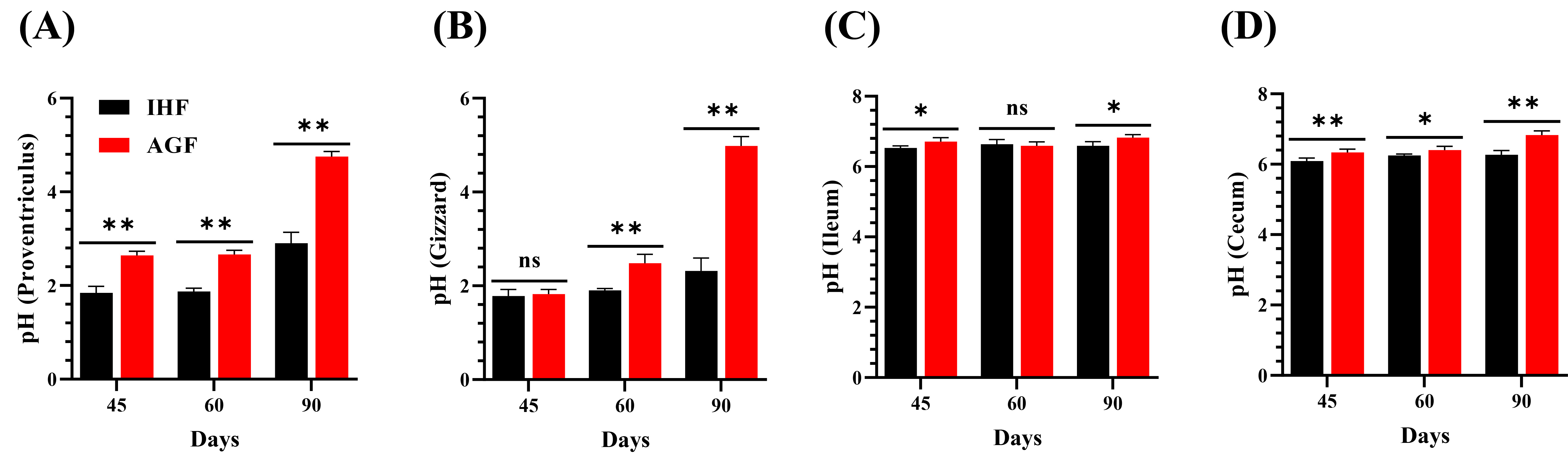

Supplement: Supplemental Figure 5 — Effects of different feeding systems on E. coli production in cecal tissues of meat geese. (A) Representative culture plate photos showing the difference between IHF and AGF meat geese in the growth of LPS-producing gram-negative E. coli. CFU/g stool. (B) E. coli cell cultures based on spectrophotometer readings at OD600 for 48h. Data with different superscript letters are significantly different (P < 0.05) according to the unpaired student T-Test. *P < 0.05, **P < 0.01. [file Image_5.jpeg]

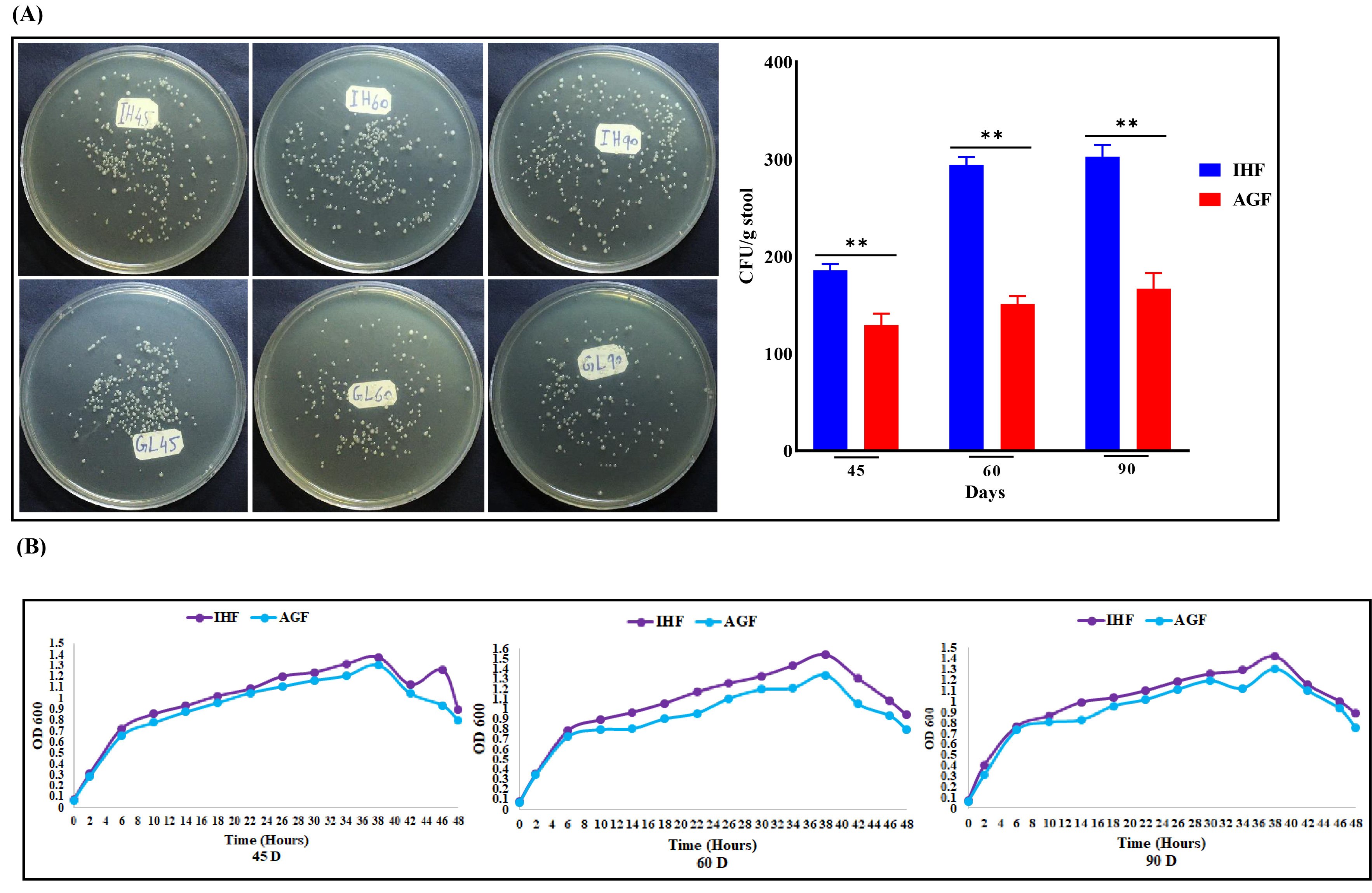

Supplement: Supplemental Figure 6 — Effects of different feeding systems on cecal morphology (100µm). VH – villus height; VW – villus width; DBV – distance between two villi; CD – crypt depth. In-house feeding system (IHF) and artificial pasture grazing system (AGF). [file Image_6.jpeg]

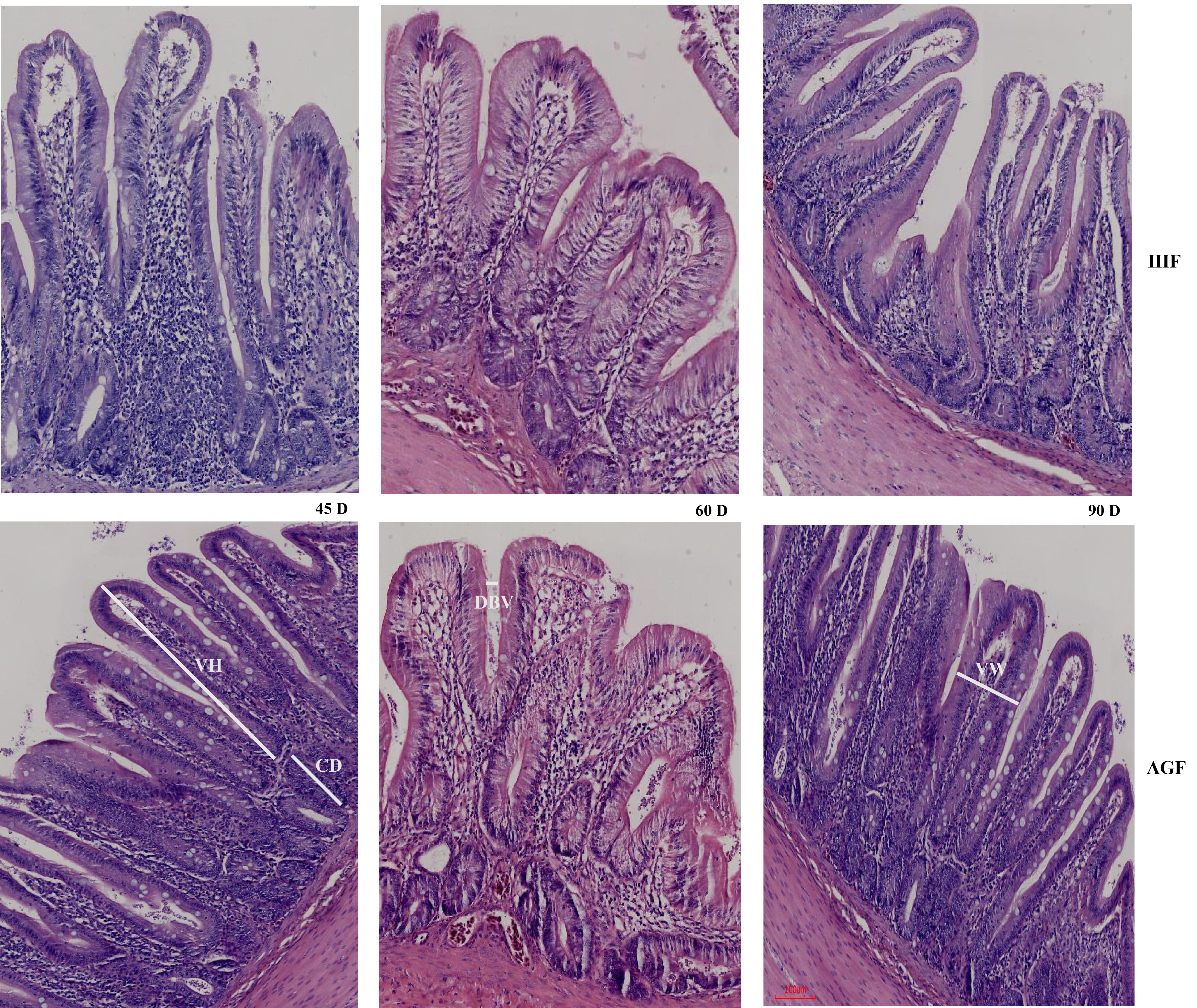

Supplement: Supplemental Figure 7 — (A) Comparison of the goblet cell number (per 20µm) of meat geese with different feeding systems. H&E staining of cecal tissues (magnification, 40×). Goblet cell (GC), In-house feeding system (IHF) and artificial pasture grazing system (AGF). Data with different superscript letters are significantly different (P < 0.05) according to the unpaired student T-Test. The asterisks symbol indicates significant differences *P < 0.05, **P < 0.01. [file Image_7.jpeg]

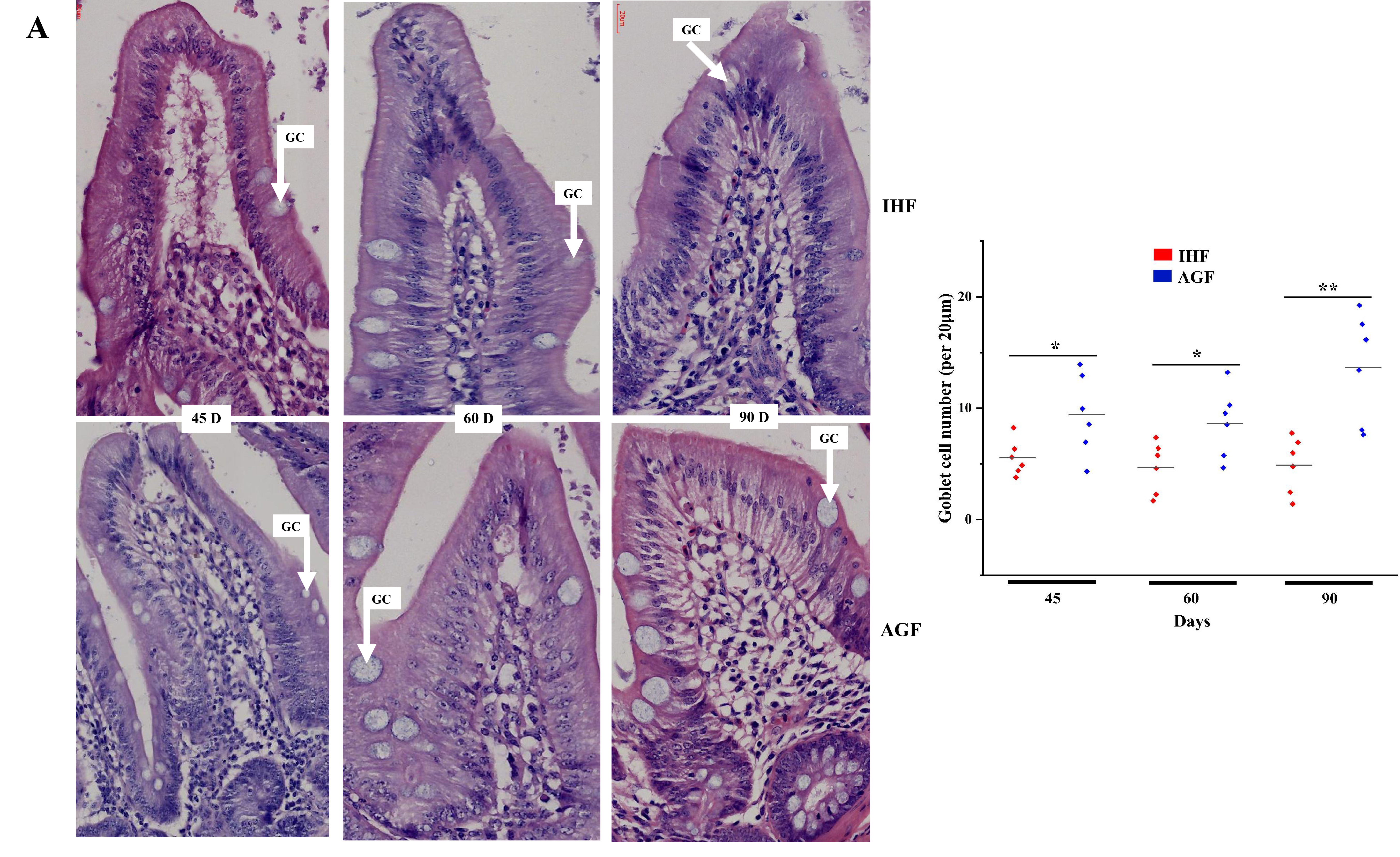

Supplement: Supplemental Figure 8 — (A) Light micrograph of the wall of cecum tissues of meat geese, hematoxylin and eosin (40µm): 1 – outer layer of muscular tonic; 2 – inner layer of muscular tonic; 3 – outer layer of lamina muscularis mucosa (LMM); 4 – submucosal nerve node; and 5 – inner layer of lamina muscularis mucosa (LMM). (B) Comparison of the cecal membrane thickness of meat geese with different feeding systems (50µm). In-house feeding system (IHF) and artificial pasture grazing system (AGF). [file Image_8.jpeg]

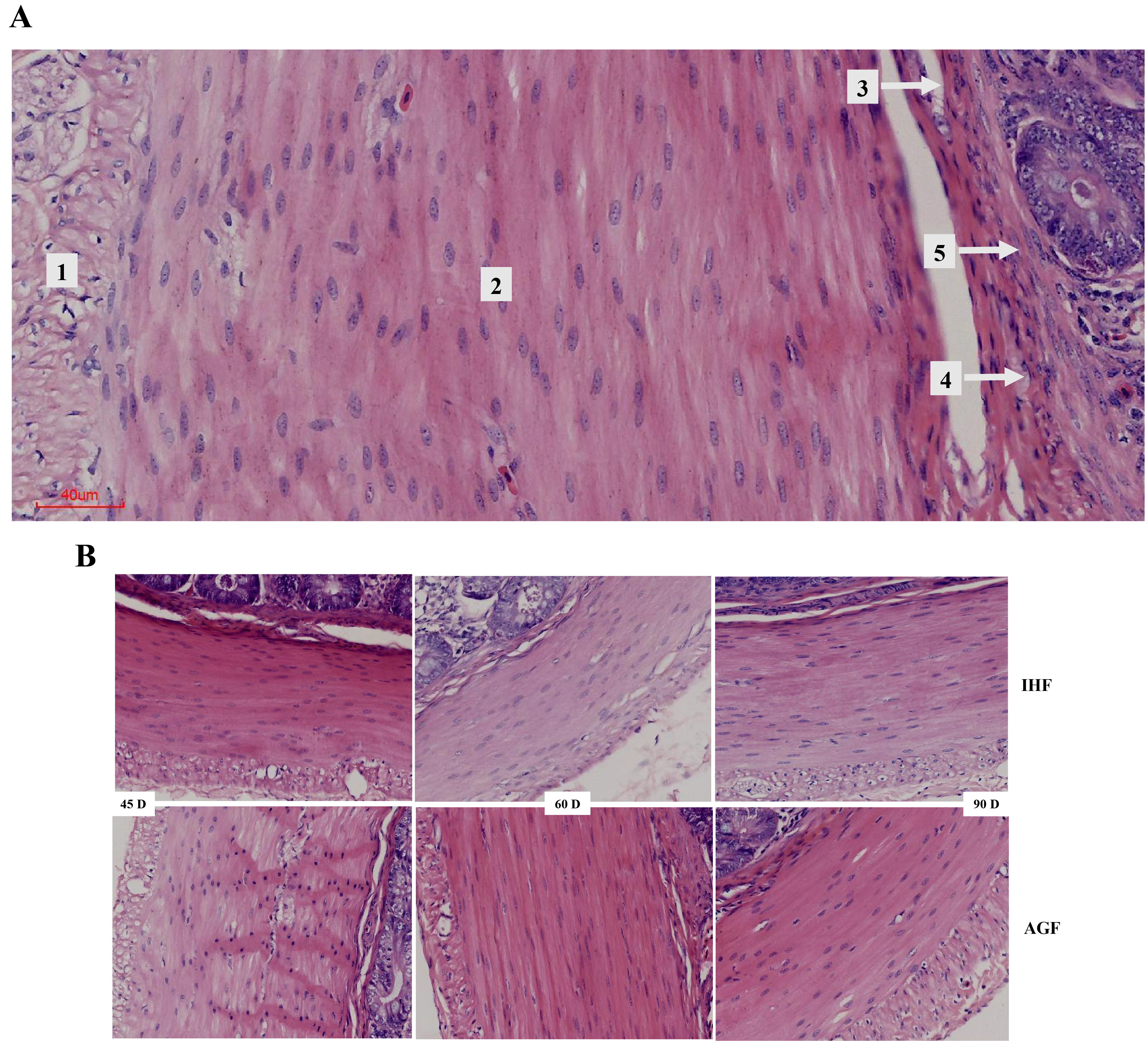

Supplement: Supplementary file 9 [file Image_9.jpeg]
