# Supplementary material for: Pasture intake protects against commercial diet-induced lipopolysaccharide production facilitated by gut microbiota through activating intestinal alkaline phosphatase enzyme in meat geese
Source: Front Immunol. 2022 Dec 8;13:1041070. doi: 10.3389/fimmu.2022.1041070 (PMC9774522; doi:10.3389/fimmu.2022.1041070)
Supplement: Supplementary file 10 [file Table_1.docx]

**Supplementary Table 1. Primer sequences used for quantitative real-time PCR**

| **Gene** | **Forward primer (Sequence 5′- 3′)** | **Reverse primer (Sequence 5′- 3′)** |
| --- | --- | --- |
| IKB-α | AAAGCTGGATGTGACCTGGA | GTTGTAGTTGGTTGCCTGCA |
| NF-KB | GGCAGAGATGGTGGAAGACT | GTTTGCCATCACCACCATGT |
| NRF2 | CGCCTTGAAGCTCATCTCAC | CCTCTCCTGCGTATATCCCG |
| iNOS | CTCATTCTCCAAGCGAACGG | GCACTCCTATCTCTGTCCCC |
| COX2 | GGTTCTACAATGGAGAGCGC | TGTTCTTGCCACTTGAGCTG |
| IL1-B | CACATCACAACCCACAGCAA | CTGCCCCTTCCGTCTTCTTA |
| IL-6 | GGAAGACCCTTGCTCTCCTT | TGGAGCCAGAAGATGAGTGG |
| TNF-α | GGTCCACAACGAGTTCATCC | AGGAGGAGGAGGAGATGGAG |
| β-actin | CAACGAGCGGTTCAGGTGT | TGGAGTTGAAGGTGGTCTCG |
| Keap1 | CTGAACGAGGCCCTCAAGTA | CAAACTCGTAGCGGGGAATG |
| MUC2 | GCCTTTCACTCAGCAGCTTT | CCATGACTCACCTGGCTGTA |
| MUC5AC | GGAAAACACGGGCTGATTGT | CTCTGACGTAGCTGGAGAGG |
| CASP3 | TCAGAGGTGACAAGTGCAGA | TCAGCACCCTACACAGAGAC |
| CASP8 | CACCACTGGCATCACAAACA | ATTCCTCCTGTCAGTCCAGC |
| NQ01 | GACCCCAAGCACTTCGTCTA | CGAAGCCTTGGATGATGACG |
| GCLC | GGAGAGTGGAATTCAGGCCT | AGAGCCACATCCATCCACAA |
| GCLM | TGCCCCACCTCCTATTGAAG | TGTGAGATCAGGTGGCATCA |
| GSTA4 | GCACCTCTGTTCCAAAGCAA | AATCCAGGACACAGCCATCA |
| IL-8 | TTCTCCTGATTTCCGTGGCT | TTCAACGTTCTTGCAGTGGG |
| CCL2 | ACTGGGAGATGTGGATGTGG | CCATACAGCTCAGCAAACCC |
| BIRC3 | TGGCCCCTGATGTTTCTCTT | AGTGTCTCCGATGCTCTGAC |
| PLAU | ACCCCTCAAAAGCTACCGAA | TCCCATGCTTCTGTTTGTGC |
| P21 | CCACGACCAGCTCCAGAAT | CTTGCCAAGACTGAGGACCT |
| P19ARF | GCTCTCAAAGGACAGCGAAC | AGGATCTGGAAGGAGCTGTG |
| TLR4 | AGGGCTACAGGTCAACAGAC | GACGTTCACCAGCCGAATAC |
| MYD88 | CTGCGTCTTTGATCGGGATG | GGCTTTGCACTTCACTGGAA |
| p16INK4α | GCCGAGCACCAGAATTTGAT | CTGCTGGGTTTTGAGGGAAC |
| lpxA | TGATGCTCCTCAGACTGCTC | GCCAACAGAGTGCTCCAAAT |
| lpxB | GAGACTTCTTCATCCGCTGC | TCCACCTGAGCAATGTCTGT |
| lpxC | GTTGTTGCAAGGAGGGTGAG | TCCAGCCTCTCACCAAACAT |
| lpxD | TCCGTTGGTCTGTCTGCTTA | GCCCTGTCTAGTGCTGAAGA |
| rfaK | TGTTCACTTCTCCTAGACAGCA | CTTGTCCTTTCAGAAGTGGCA |
| rfaL | CCTCTTCTGCTGGCACTACT | TTCCCATCCCCAGCAGAAAT |
| LBP | CTGTCTGTGGCTCTGATGGA | AGCACCGAACCAGTCCTTTA |
| sCD14 | TGGATGGGGCTGTGGTATAC | GTCACAGGTCAGCTCTCGAT |
| ALPi | CCTAGGGATGAAGCAGGGAG | TGGGCCACTGTCATCTCATT |
| CG5150 | ACGAGCAGAACTACATCCCC | TAGCAGAGGAGGAGGAGGAG |
| CG10827 | GAGGGCAAAGAATGGGCAAA | ACCAAGAAAACAGCAGCCAG |
| E-cadherin | GGTGACGGTGGAGAACAAAG | CTGGGCTGTGTAGGATGTGA |
| dlg1 | AGTGATATTGACCGTGGCCA | AATCACGTCCATCCACCTCA |
| IL-4 | TGACAGGGTATTGGTCCACC | ACGGAAGAAGCAGAAGGTGA |
| IL-10 | CATCAAGAACAGCGAGCACC | GCACCCACCTTTTCAAACGT |
| LC8 | GAGGAGATGCAGCAGGACTC | GAGTCACGTAGCTGCCAAAG |
| Cytochrome C | GGGTTACATAGGGCGGAGTC | GCCTCCCTTCTCAACCGTAT |
| Z0-1 | CCACCTCTGTCCAGCTCTTC | CACCGGAGTGATGGTTTTC |
| Occludin | ACTCCTCCAATGGACAAGTG | CCCCACCTGTCGTGTAGTCT |
| Claudin | GTCATCGCCCATCAGAAGAT | ACTGTTGGACAGGGAACCAG |
