# Supplementary material for: Pasture intake protects against commercial diet-induced lipopolysaccharide production facilitated by gut microbiota through activating intestinal alkaline phosphatase enzyme in meat geese
Source: Front Immunol. 2022 Dec 8;13:1041070. doi: 10.3389/fimmu.2022.1041070 (PMC9774522; doi:10.3389/fimmu.2022.1041070)
Supplement: Supplementary file 11 [file Table_2.docx]

**Supplementary Table 2. Effect of artificial pasture grazing system on pH of proventriculus, gizzard, ileum, and cecum of meat geese.** Data expressed as mean ± SEM

| **Age, d** | **Parameters** | **IHF** | **AGF** | **P-value** |
| --- | --- | --- | --- | --- |
| 45 d | Proventriculus | 1.84±0.14 | 2.64±0.09 | 2.3704E-05 |
| 60 d |  | 1.87±0.08 | 2.66±0.1 | 7.28674E-06 |
| 90 d |  | 2.9±0.24 | 4.75±0.11 | 1.10084E-05 |
| 45 d | Gizzard | 1.78±0.14 | 1.82±0.11 | 0.330742017 |
| 60 d |  | 1.9±0.04 | 2.48±0.21 | 0.001 |
| 90 d |  | 2.32±0.27 | 4.98±0.2 | 1.58863E-09 |
| 45 d | Ileum | 6.53±0.06 | 6.71±0.11 | 0.023 |
| 60 d |  | 6.64±0.13 | 6.59±0.10 | 0.41 |
| 90 d |  | 6.59±0.11 | 6.82±0.09 | 0.02 |
| 45 d | Cecum | 6.09±0.1 | 6.34±0.1 | 0.001 |
| 60 d |  | 6.25±0.04 | 6.4±0.12 | 0.01 |
| 90 d |  | 6.27±0.13 | 6.83±0.13 | 1.01066E-05 |
