# Supplementary material for: Pasture intake protects against commercial diet-induced lipopolysaccharide production facilitated by gut microbiota through activating intestinal alkaline phosphatase enzyme in meat geese
Source: Front Immunol. 2022 Dec 8;13:1041070. doi: 10.3389/fimmu.2022.1041070 (PMC9774522; doi:10.3389/fimmu.2022.1041070)
Supplement: Supplementary file 12 [file Table_3.docx]

**Supplementary Table 3. Effects of artificial pasture grazing system on the cecal morphology of meat geese.** Data expressed as mean ± SEM.

| **Parameters** | **45 D** | |  | **60 D** | |  | **90 D** | |  | **P-values** | | |
| --- | --- | --- | --- | --- | --- | --- | --- | --- | --- | --- | --- | --- |
|  | **IHF** | **AGF** |  | **IHF** | **AGF** |  | **IHF** | **AGF** |  | **45 D** | **60 D** | **90 D** |
| Villus height (um) | 63.69±10.22 | 96.89±16.96 |  | 62.19±12.74 | 112.96±21.23 |  | 68.93±12.64 | 93.71±17.14 |  | <0.001 | <0.0003 | <0.01 |
| Villus width (um) | 32.97±3.69 | 41.84±9.86 |  | 25.97±7.48 | 37.31±9.56 |  | 23.37±6.59 | 41.54±6.6 |  | <0.033 | <0.023 | <0.0004 |
| Surface area (um^2^) | 4379.64±969.12 | 11776.67±5400.16 |  | 3762.17±1454.98 | 7901.81±2201.51 |  | 3960.01±596.81 | 10442.38±2385.9 |  | <0.004 | <0.0016 | <0.0004 |
| Crypt depth (um) | 24.82±9.32 | 15.5±1.61 |  | 24.12±5.57 | 16.33±6 |  | 25.4±5.42 | 17.37±3.42 |  | <0.018 | <0.02 | <0.01 |
| Villus height/Crypt depth | 2.13±0.17 | 1.86±0.73 |  | 1.64±0.37 | 1.56±0.35 |  | 1.38±0.4 | 1.72±0.54 |  | 0.2 | 0.37 | 0.1 |
| Distance between villi (um) | 3.37±0.42 | 6.02±0.88 |  | 3.12±0.44 | 6.46±0.59 |  | 3.51±0.99 | 6.51±1.16 |  | <0.00003 | <3E-07 | <0.005 |
