# Supplementary material for: Pasture intake protects against commercial diet-induced lipopolysaccharide production facilitated by gut microbiota through activating intestinal alkaline phosphatase enzyme in meat geese
Source: Front Immunol. 2022 Dec 8;13:1041070. doi: 10.3389/fimmu.2022.1041070 (PMC9774522; doi:10.3389/fimmu.2022.1041070)
Supplement: Supplementary file 13 [file Table_4.docx]

| **Age, d** | **IHF** | **AGF** | **P-value** |
| --- | --- | --- | --- |
| 45 | 20.8±8.73 | 7.58±2.64 | 0.003 |
| 60 | 14.25±4.88 | 7.11±1.6 | 0.01 |
| 90 | 10.84±2.32 | 6.93±0.76 | 0.001 |

**Supplementary Table 4. Effect of different feeding systems on the numbers of apoptotic cells per field in the villus of cecal tissues.** In-house feeding system (IHF) and artificial pasture grazing system (AGF). Data expressed as mean ± SEM.
