# Supplementary material for: Pasture intake protects against commercial diet-induced lipopolysaccharide production facilitated by gut microbiota through activating intestinal alkaline phosphatase enzyme in meat geese
Source: Front Immunol. 2022 Dec 8;13:1041070. doi: 10.3389/fimmu.2022.1041070 (PMC9774522; doi:10.3389/fimmu.2022.1041070)
Supplement: Supplementary file 14 [file Table_5.docx]

| **Parameters** | **45 D** | |  | **60 D** | |  | **90 D** | |  | **P-value** | | |
| --- | --- | --- | --- | --- | --- | --- | --- | --- | --- | --- | --- | --- |
|  | **IHF** | **AGF** |  | **IHF** | **AGF** |  | **IHF** | **AGF** |  | **45 D** | **60 D** | **90 D** |
| Inner layer (um) | 48.81±8.64 | 61.39±6.06 |  | 47.42±21.93 | 80.35±8.67 |  | 39.79±17.75 | 68.34±13.34 |  | <0.00765 | <0.003 | <0.005 |
| Outer layer (um) | 8.54±2 | 10.88±1.41 |  | 7.84±1.69 | 17.25±1.08 |  | 8.49±2.18 | 20.26±4.31 |  | <0.02 | <2.18E-07 | <6.90E-05 |
| Total (um) | 57.35±10.2 | 72.26±5.37 |  | 55.26±22.19 | 97.6±9.02 |  | 48.27±17.3 | 88.6±14.27 |  | <0.005 | <0.001 | <0.001 |
| Relative thickness of muscular tonic (um) | 24.84±4.51 | 44.49±3.26 |  | 12.88±4.84 | 29.33±2.7 |  | 9.07±3.46 | 20.44±4.37 |  | <2.94E-06 | <1.34E-05 | <0.0003 |
|  |  |  |  |  |  |  |  |  |  |  |  |  |
| Inner layer (um) | 2.71±0.32 | 3.87±0.71 |  | 3.13±1.27 | 7.75±2.55 |  | 3.1±0.88 | 4.98±1.47 |  | <0.002 | <0.001 | <0.01 |
| Outer layer (um) | 1.08±0.28 | 1.59±0.25 |  | 1.27±0.28 | 1.53±0.19 |  | 1.06±0.28 | 1.48±0.22 |  | <0.004 | <0.04 | <0.01 |
| Total (um) | 3.78±0.36 | 5.47±0.59 |  | 4.39±1.16 | 9.28±2.52 |  | 4.16±0.93 | 6.46±1.51 |  | <0.0001 | <0.001 | <0.005 |
| Relative thickness of muscularis mucosa (um) | 1.64±0.15 | 3.39±0.55 |  | 1.03±0.31 | 2.79±0.77 |  | 0.78±0.21 | 1.5±0.45 |  | <0.00001 | <0.0002 | <0.003 |

**Supplementary Table 5. Effect of different feeding systems on the thickness of ileal muscular tonic and muscularis mucosa (50µm).** In-house feeding system (IHF) and artificial pasture grazing system (AGF). Data expressed as mean ± SEM.
